# Effect of Different Surface Conditions on Toughness of Vanadis 6 Cold Work Die Steel—A Review

**DOI:** 10.3390/ma12101660

**Published:** 2019-05-22

**Authors:** Peter Jurči

**Affiliations:** Faculty of Materials Science and Technology in Trnava, Slovak University of Technology in Bratislava, Jána Bottu 2781/25, 917 24 Trnava, Slovakia; p.jurci@seznam.cz

**Keywords:** Vanadis 6 die steel, surface finish, nitriding, PVD coating, toughness, fractography

## Abstract

The effects of surface roughness, presence of nitrided diffusion regions, and magnetron sputtering of Cr_2_N–6Ag thin films on the toughness of Cr–V ledeburitic Vanadis 6 die steel were investigated by using the flexural strength measurement method, which was coupled with careful microstructural investigations and analyses of fractured surfaces. The results undoubtedly show that enhanced surface roughness reduces the material toughness, since the cusps formed on the metallic surface as a result of the machining act as preferential sites for crack nucleation and growth. The presence of nitrided regions on the surface, on the other hand, forms a structural notch there, which has a strong detrimental effect on toughness. Deposition of Cr_2_N–6Ag thin films has only marginal effect on the steel toughness. Practical recommendations for the designers, heat treaters, and coaters of the tools are thus that they should maintain the surface finish quality of the tools as high as possible, avoid too thick and supersaturated nitrided regions, and that there is almost no risk of tool embrittlement due to physical vapor deposition (PVD) coating.

## 1. Introduction

High-carbon, high-chromium, and high-vanadium cold work tool steels have been frequently used in modern industries in applications where superior wear resistance and strength are required. This includes industrial branches like powder compacting, paper cutting, sheet forming, and fine blanking.

The high strength and hardness, and excellent wear resistance of these tool steel grades are a result of the standard heat treatment procedure. It comprises vacuum austenitizing, holding at the desired temperature for a pre-determined duration, and inert gas quenching, followed by immediate tempering. Properly performed heat treatment provides the steels with a hardness of around 60 HRC at the secondary hardness peak. Excellent wear resistance is ensured by a high amount of carbides, among which the MC particles play a dominant role. 

However, ledeburitic steels generally have low toughness. Moreover, these materials suffer from poor and inhomogeneous carbide distribution when manufactured by classical ingot metallurgy. This causes an “extra” embrittlement of the materials and a great level of anisotropy of the mechanical properties [1]. Advanced powder metallurgy (PM) ledeburitic steels do not manifest anisotropy of the mechanical properties [2]. Despite that, their toughness (being represented by flexural strength) is limited by approx. 4000 MPa, and the fracture toughness is around 15–16 MPa·m^1/2^ [3], at a hardness of 60–61 HRC.

After the heat treatment, the tools should be surface finished before their use. Surface finishing involves machining operations like fine grinding, lapping, and polishing. These operations provide the tools with the final surface roughness, which is specific for each of them. Fine grinding, for instance, results in surface roughness (R_a_) of 0.2–0.4 μm, while lapping reduces the R_a_ to a level of 0.02 μm, and polishing provides the metallic surface with a so-called “mirror finish”. 

It is well known that the surface roughness may have important impacts on the toughness of brittle materials like dental ceramics, as pointed out by Vasconcellos Amarante et al. [4] and by Hallmann et al. [5]. One can expect that the toughness of heat-treated ledeburitic tool steels will also be influenced by surface roughness, as they belong to the group of relatively brittle materials. Despite that, the information on the effect of surface roughness on fracture performance of ledeburitic steels is lacking, and there is no comprehensive study on this effect published yet. Only Spies, Riese, and Hoffmann [6] reported that the flexural strength of hardened and tempered M2 high-speed steel decreased from 3780 to 3700 and 3600 MPa when the surface roughness increased from 0.1 to 0.5 and 1 μm, respectively.

Plasma nitriding has been widely used in steels, resulting in benefits such as the increase of fatigue life [7], the improvement of tribological properties [8,9], and the increase of corrosion resistance [10]. The plasma nitriding applied to tool steels has recently gained a new impulse with the development of duplex coating, which is a combined treatment consisting of the deposition of a hard physical vapor deposition (PVD) layer on a pre-nitrided surface [11,12,13,14,15,16]. The presence of an intermediate hardened layer under very hard thin films has shown benefits for the mechanical properties of the surface. Bell et al. [17], for instance, suggested a better load bearing capacity, improved fatigue resistance, and a smooth hardness profile from the surface to the substrate, resulting in lower levels of residual stresses in the layer/substrate interface. It was also demonstrated that the duplex coating leads to much better adhesion of thin films to the substrates [12,13,16] and higher wear resistance [12,14], and thereby leads to manyfold extended tool service life [11,15,18].

All the cited works show the benefits of nitriding the surfaces of tool steels. Nevertheless, nitrogen input to the surface makes the steel harder and therefore more brittle. Embrittlement of ledeburitic tool steels has been indicated by Hock et al. [19], and later experimentally proved by Kwietniewski et al. [20] by industrial testing of nitrided and duplex-coated single-point turning tools. Unfortunately, the embrittlement has not been determined exactly but only estimated based on evaluation of tools damage as well as by microstructural evaluation of nitrided surfaces. Hence, an exact assessment of the effect of a nitrided surface on the bulk toughness is not available in the scientific literature to date.

Thin ceramic films based on transition metal nitrides (carbides, oxides) have been proven to be good candidates for protecting the base tool steel against wear, corrosion, and other unwanted damage.

In the field of thin solid films, PVD (physical vapor deposition)-produced titanium nitride (TiN) is still the most widely accepted in engineering applications. The combination of high hardness, wear resistance, chemical inertness, and low friction coefficient characteristics in TiN makes it attractive as a tribological coating. However, the main drawback of TiN is its limited oxidation resistance (approximately 500 °C). The resistance against high-temperature oxidation can be improved by the addition of Al and Cr [21,22]. TiAlN coatings have been developed as alternatives to TiN because of their higher corrosion resistance (up to 750 °C) and higher hardness [23]. But their wear performance at ambient temperature is negatively influenced due to their high friction coefficient [24]. 

Another alternative to TiN are the Cr_x_N_y_ films, which have been developed over the past three decades. They have gained early popularity in a variety of industrial applications due to superior wear resistance [25,26], good corrosion resistance [27,28,29], and good cutting properties in copper machining [30], aluminium die casting [29], or in woodworking [31,32]. Cr_x_N_y_ films can be synthesized in wide ranges of chemistries, phase constitutions, and properties. The microhardness of Cr_x_N_y_ films ranges between 1500 and 3000 HV [30,33,34,35,36]. The Young modulus can also be varied, from the lowest values of around 188 to the highest ones of 315 GPa [33,35]. The adhesion of Cr_x_N_y_ thin films can be influenced by their phase constitution [37] and internal stress level [37], but also by proper selection of the substrate. Adhesion is better when hard substrates (ledeburitic steels, cemented carbides) are coated [32,34,38]. Later, it was found that small additions of silver can provide the Cr_x_N_y_ films with self-lubricating properties and very low friction coefficients, with almost unaffected or rather slightly improved nanohardness and adhesion [39,40,41,42]. Despite the high universality of Cr_x_N_y_ films, their use is not possible in selected cases. Carlsson and Olsson [43], for instance, concluded that metal–carbide-doped diamond-like carbon (DLC) coatings are good candidates for tool coatings in the dry cold forming of hot dip Zn steel sheets. These steel sheets are not suited for dry forming due to severe material pick-up, and the use of Cr_x_N_y_ coating fails in these cases due to high friction coefficients and material transfer from the steel sheet surfaces. 

Even though a great number of scientific papers have been published about the growth, microstructure, and key properties of PVD thin films, only a little attention has been paid to the effect of the presence of thin ceramic films on the substrate toughness. 

To the best knowledge of the author, no comprehensive and overview report has been made so far to review the influence of different surface treatments on the toughness of ledeburitic steels. The major aim of the present overview is to overcome this limitation and to summarize, review, and discuss the existing information about the effect of different surface states on the toughness of powder metallurgy cold work die steel Vanadis 6. The impacts of different surface roughness developed by machining operations, presence of plasma-nitrided regions with different thickness and microhardness, and influence of CrAgN nanocomposite thin films on the flexural strength (toughness measure) of the experimental material will be presented, analyzed, and discussed. In the text, the original papers will be referred to in some places in order to avoid potential confusions.

## 2. Description of Experimental Material, Processing, and Investigations Methods

A commercially available PM ledeburitic cold work tool steel Vanadis 6 (Uddeholm AB, Hagfors, Sweden) with a nominal composition (wt.%) of 2.1% C, 1.0% Si, 0.4% Mn, 6.8% Cr, 1.5% Mo, 5.4% V, and Fe as the balance was used as an experimental material. The initial state of the material was soft-annealed, with a hardness of 284 HV10. The microstructure of the steel manifests a high degree of isotropy. Hence, the orientation of the semi-finished product was disregarded in sampling [2].

**Figure 1:**
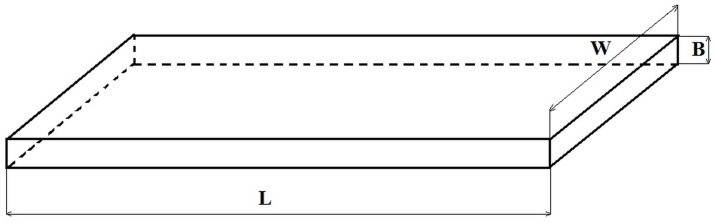
A schematic of the specimens used for flexural strength determination.

Two types of specimens were manufactured. The first ones were cylinders with 20 mm in diameter and 6 mm in thickness for microstructural examinations, and the second group of specimens was plates with dimensions (thickness × width × length) of 1 × 10 × 100 mm (Figure 1) for flexural strength determination. All the specimens were milled to a surface roughness of 6 μm and subjected to the pre-determined heat treatment schedule. The heat treatment consisted of the following steps: Gradual heating up to the desired austenitizing temperature *T*_A_ (1050 °C) in a vacuum furnace and then holding at that temperature for 30 min to homogenize the austenite, which was followed by quenching by nitrogen gas (5 bar pressure). Immediately after quenching to the room temperature, the specimens were moved to a tempering furnace. Double tempering was carried out at a temperature of 530 °C, with a duration of each tempering cycle of 2 h. The material was cooled down slowly to the room temperature after each tempering cycle.

Then, the specimens were divided to several batches (Table 1).

Plasma nitriding was performed in a RUBIG PN 60/60 (Rubig GmBH, Wels, Austria) device at different temperatures and for various processing durations (Table 2). Five specimens (from Batch 4; Table 1) were treated at each of processing parameter combinations. First, the specimens were cleaned and degreased in an ultrasonic acetone bath for 15 min. Then, they were moved to the plasma nitriding device, where they were heated up to the pre-determined temperature, and sputter cleaned for 30 min in a pure hydrogen atmosphere in order to activate the surface. Afterwards, an atmosphere containing nitrogen and hydrogen in a ratio of 1:3, at a pressure of 300 Pa, was introduced into the processing chamber. The plasma nitriding procedures were carried out by using a voltage of 500 V, in a pulse regime with the pulse time of 100 μs. After the plasma nitriding, the specimens were cooled down slowly to the room temperature.

Cr_2_N–6Ag thin films were synthesized in a Hauzer-Flexicoat 850 (Hauzer, Venlo, The Netherlands) magnetron sputter deposition system, in a pulse regime with a frequency of 40 kHz. For the growth of the Cr_2_N–6Ag films, two water-cooled cathodes, the first one made of chromium with the standard purity of 99% and the second one manufactured out of silver with the purity of 99.98%, were used. The cathodes were positioned opposite to one another, at an angle of 45° with respect to the substrate surface. The output power of the Cr cathode was 5.8 kW, and that of the Ag cathode was 0.2 kW, in order to add 6 mass% of Ag into the base Cr_2_N.

The substrates were placed between the targets on rotating holders with a rotation speed of 3 rpm. At the beginning of the process, pure Ar (99.999% purity) was introduced into the processing chamber in order to sputter clean the substrates. During the cleaning step, the substrate temperature reached 250 °C, the negative substrate bias was 200 V, and the duration of sputter cleaning was 15 min. Just prior to deposition, both targets were sputter cleaned for 7 min, with the samples covered with a protective shutter during this period. Next, pure N_2_ (99.998% purity) was introduced into the chamber to obtain an atmospheric composition of N_2_:Ar = 1:4.5 and a pressure of 0.15 mbar. A negative substrate bias of 100 V was used for the growth of the films. The deposition temperature was increased to 500 °C by the use of internal wall resistive heaters.

The microstructure of the examined steel was evaluated by light microscopy, by using a Neophot 32 apparatus (Carl Zeiss AG, Oberkochen, Germany). The same light microscope was used for the inspection of the surface morphologies of the specimens with different roughness as well as for microstructural investigations of differently plasma nitrided specimens. The specimens were Nital (3% ethanol solution of nitric acid) etched after standard metallographic preparation. 

For more precise examinations, however, scanning electron microscopy (SEM) and transmission electron microscopy (TEM) were used. A JEOL JSM 7600 F (Jeol Ltd., Tokyo, Japan) apparatus equipped with an energy-dispersive spectroscopy (EDS) detector (Oxford Instruments, plc., High Wycombe, UK) and a wavelength-dispersive spectrometry (WDS) detector was used for SEM observations. The microstructure was recorded in the secondary electron (SE) detection regime. The microstructure of magnetron-sputtered thin films was examined on the fractured surfaces of specimens prepared as follows: After deposition, the specimens were immersed into liquid nitrogen, held there for 15 min, and broken down. Then, they were reheated slowly to room temperature, cleaned ultrasonically in acetone, and dried before inserting into the SEM. TEM was carried out by using a JEOL 200CX microscope (Jeol Ltd., Tokyo, Japan) operating at an acceleration voltage of 200 kV. Thin foils were made by a standard preparation technique. Pieces with square cross-sections and thickness of 0.15 mm were cut off from the material, and mechanically thinned to a thickness of approximately 20 μm. The final thinning was realized by using an electrolytic jet-polisher (Tenupol 5).

Microhardness depth profiles throughout the nitrided regions and the base material microhardness were measured with a Buehler Indentament 1100 tester (Buehler Ltd., Lake Bluff, IL, USA), at a load of 50 g (HV 0.05) and loading time of 20 s.

The nanohardness and the Young´s modulus (E) of Cr_2_N–6Ag thin films were measured by using a NanoTest (Micro Materials Ltd, Wrexham, UK) nanohardness tester. The maximum normal load was 20 mN, and a Berkovich indenter was employed. Ten measurements were made, and the mean value and the standard deviation were then calculated. The penetration depth (and loading) was chosen to not exceed one tenth of the total coating thickness.

In order to analyze the chemical composition throughout the Cr_2_N–6Ag thin films, Glow Discharge Optical Emission Spectroscopy (GDOES) by using a Spectruma GDA 750 (Spectruma Analytik GmbH, Hof, Germany) device was carried out.

Flexural strength was determined by using an Instron 8862 testing device (Instron, Norwood, MA, USA), with the distance between loading roller supports of 80 mm, at an ambient temperature. Specimens were loaded in three-point bending at a loading rate of 1 mm/min, up to the moment of fracture. Flexural strength was determined from the maximum (fracture) load according to a standard approach. In addition, the total work of fracture (*W*_of_) was determined as deformation energy evaluated from corresponding area below the measured load–deflection (load–displacement) curve.

The flexural strength was calculated using the formula:

(1) $$R_{mo}=\frac{3\times F\times l_{o}}{2\times B^{2}\times W}$$

where *R_mo_* is the flexural strength, *F* is the load at the fracture point, *L_o_* is the distance between supports, and *B* and *W* are the thickness and width of the specimen, respectively.

The fracture surface morphology was investigated by a JEOL JSM 7600F scanning electron microscope.

## 3. Results, Discussion, and Practical Recommendations

### 3.1. Microstructure of Examined Steel

**Figure 2:**
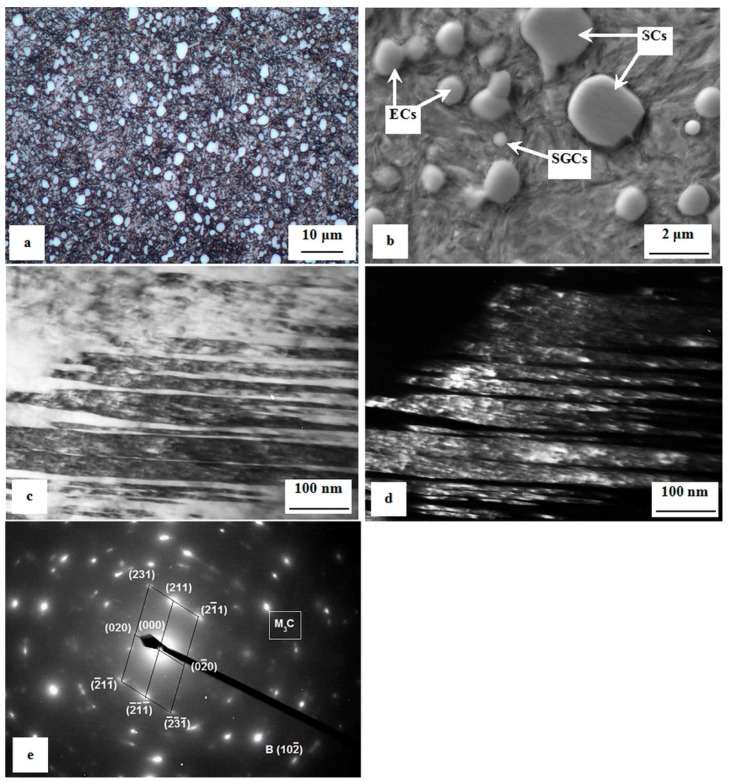
Microstructure of the experimental material after the given heat treatment schedule. (**a**) Optical micrograph, (**b**) SEM micrograph, (**c**) bright-field TEM micrograph showing internally twinned martensitic laths, (**d**) corresponding dark-field micrograph showing precipitates inside the martensitic domain, and (**e**) diffraction patterns of the precipitates.

Figure 2 is a compilation of differently acquired micrographs showing the microstructure of the examined steel after realization of the heat treatment schedule. An optical micrograph, shown in Figure 2a, demonstrates that the microstructure of the steel is composed of tempered martensite and undissolved carbides, which are uniformly distributed throughout the martensitic matrix. The sizes of the carbides range from several hundreds of nm up to approx. 3 μm. A more detailed SEM micrograph, shown in Figure 2b, enables us to distinguish between eutectic carbides (ECs), secondary carbides (SCs), and small globular carbides (SGCs), according to the classification published recently [44]. The martensite manifests clear lath morphology, with no presence of retained austenite as this phase is decomposed by high temperature tempering. A bright-field TEM micrograph, shown in Figure 2c, shows that many of the martensitic laths are internally twinned. The widths of the twins are of around 20–30 nm. There are a lot of precipitates located inside the twins. This is sufficient for obtaining the dark-field image, shown in Figure 2d, as well as for obtaining a good intensity of reflections in the corresponding diffraction patterns, as shown in Figure 2e. Analysis of the reflections disclosed that the particles are of cementitic nature.

The as-tempered hardness of the material was 733 ± 9 HV10, and the fracture toughness was determined to be 15.72 ± 0.31 MPa·m^1/2^ [3].

### 3.2. Effect of Surface Roughness

**Figure 3:**
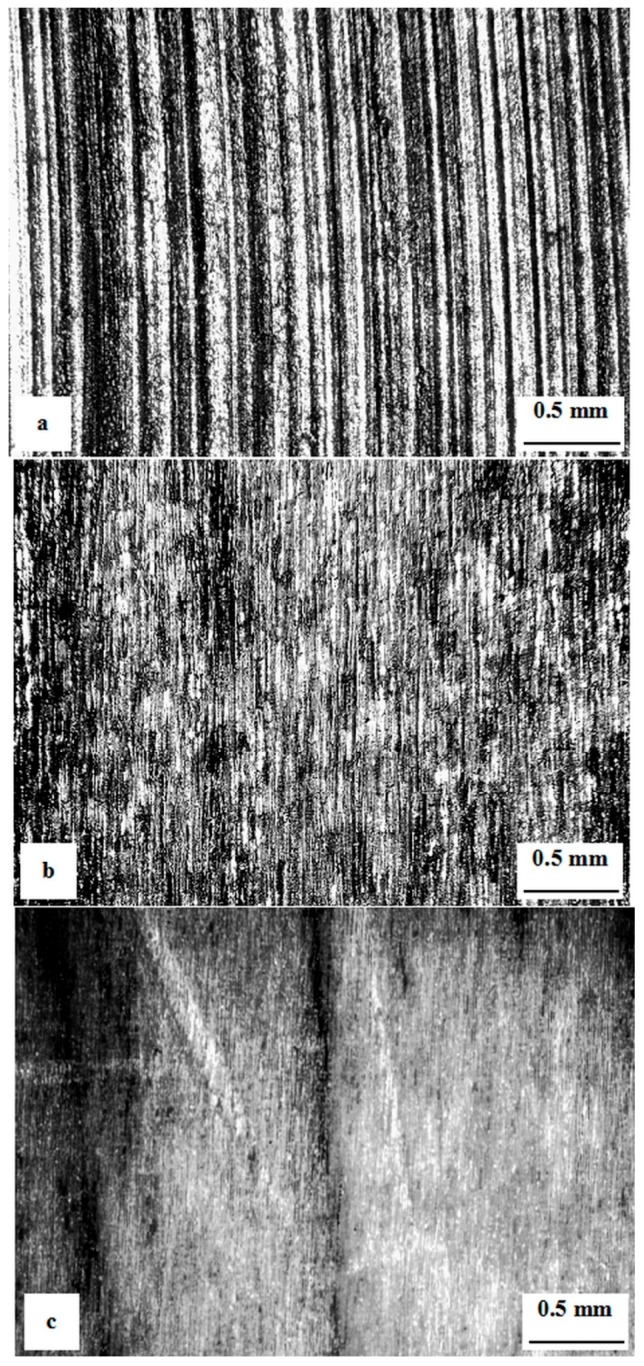
Plan-view optical micrograph showing the surfaces of specimens made of Vanadis 6 steel with different roughness: (**a**) Milled, (**b**) fine ground, and (**c**) polished. Adapted from the corresponding literature [45]. (Copyright, Materials Engineering/Materiálové inžinierstvo, 2011).

The effect of surface roughness on toughness of the Vanadis 6 steel was investigated in our recent work [45]. Figure 3 shows plan-view optical micrographs showing the surface finishes of the tested specimens. After milling, the surface roughness was 6 μm (Figure 3a). Subsequent grinding led to a substantial reduction of surface roughness, to a value of 0.3 μm (Figure 3b). The third batch of the specimens was subjected to mirror polishing, and the surface was then very smooth (Figure 3c). 

**Figure 4:**
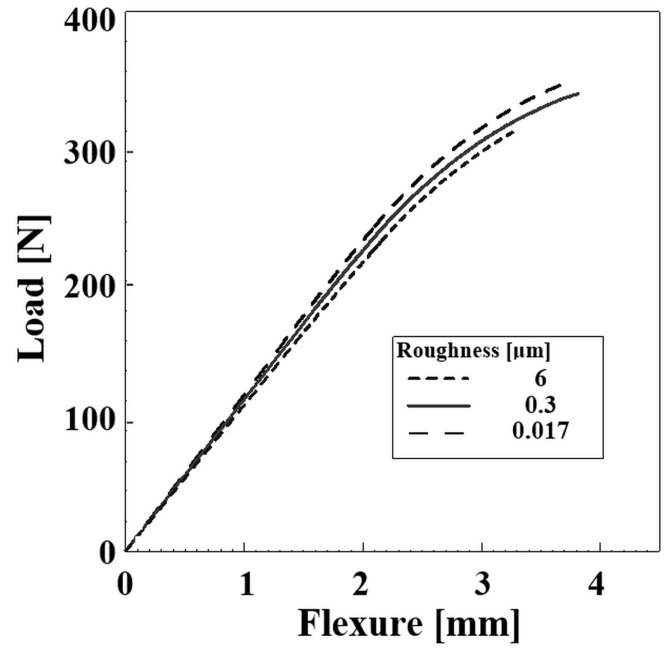
Load–flexure diagrams of the specimens with different surface roughness.

The load–flexure diagrams obtained by the flexural strength testing of specimens with different surface roughness are shown in Figure 4. It is shown that all the curves manifest the load–flexure dependence typical for hard and relatively brittle steels, i.e., there is linear dependence of these two quantities evident up to relatively high loading, which is followed by a small region suggesting limited plastic behavior of the material at higher loading. Alternatively, there is also an apparent difference between the load–flexure traces visible on the diagram—the load at the fracture moment decreases with increasing surface roughness.

**Figure 5:**
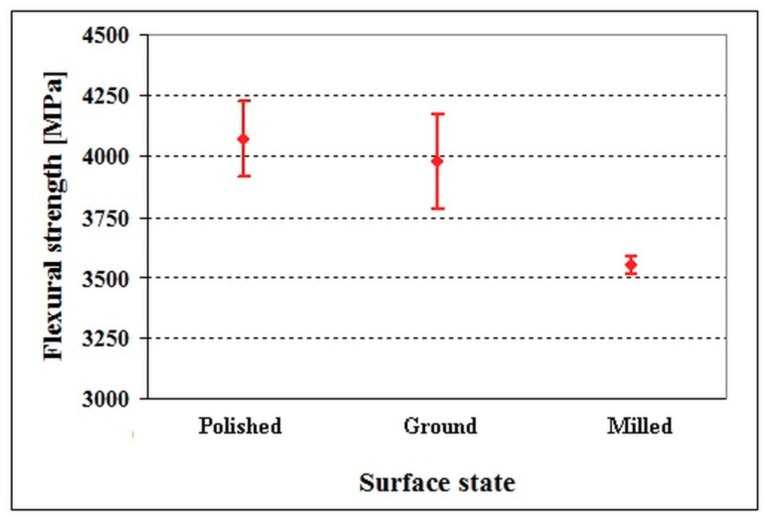
Flexural strengths of specimens made of the Vanadis 6 steel with different surface finish. Adapted from the corresponding literature [45]. (Copyright, Materials Engineering/Materiálové inžinierstvo, 2011).

Figure 5 shows the dependence of flexural strength on the surface finish. It is shown that the flexural strengths for both the polished specimens (R_a_ = 0.017 μm) and the fine ground ones (R_a_ = 0.3 μm) were of around 4000 MPa, and the differences lie within the range of statistical uncertainty of the obtained results, even though the polished surface state seems to be more convenient from the point of view of high-material toughness maintenance (due to its slightly higher mean value as well as smaller scatter of the obtained results). Alternatively, the flexural strength of the milled material (R_a_ = 6 μm) manifests a clear decrease.

**Figure 6:**
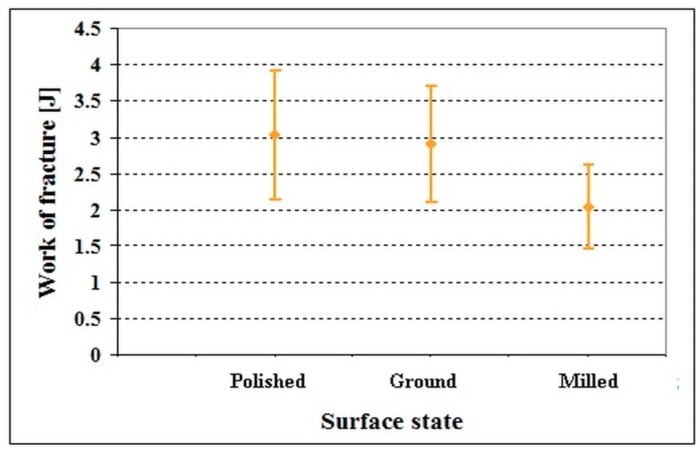
Work of fracture of specimens made of the Vanadis 6 steel with different surface finish. Adapted from the corresponding literature [45]. (Copyright, Materials Engineering/Materiálové inžinierstvo, 2011).

The differences in flexural strength values, shown in Figure 5, are reflected in the values of the work of fracture, shown in Figure 6. As shown here, the work of fracture of polished material is almost the same as that of ground steel, and the differences lie within the range of statistical uncertainty of the results. On the other hand, the work of fracture of milled steel is lower despite its statistical uncertainty range considerably overlapping with those calculated for both the polished and ground specimens. In other words, the dependence of the work of fracture on the surface finish closely follows the flexural strength dependence on the same surface parameter.

**Figure 7:**
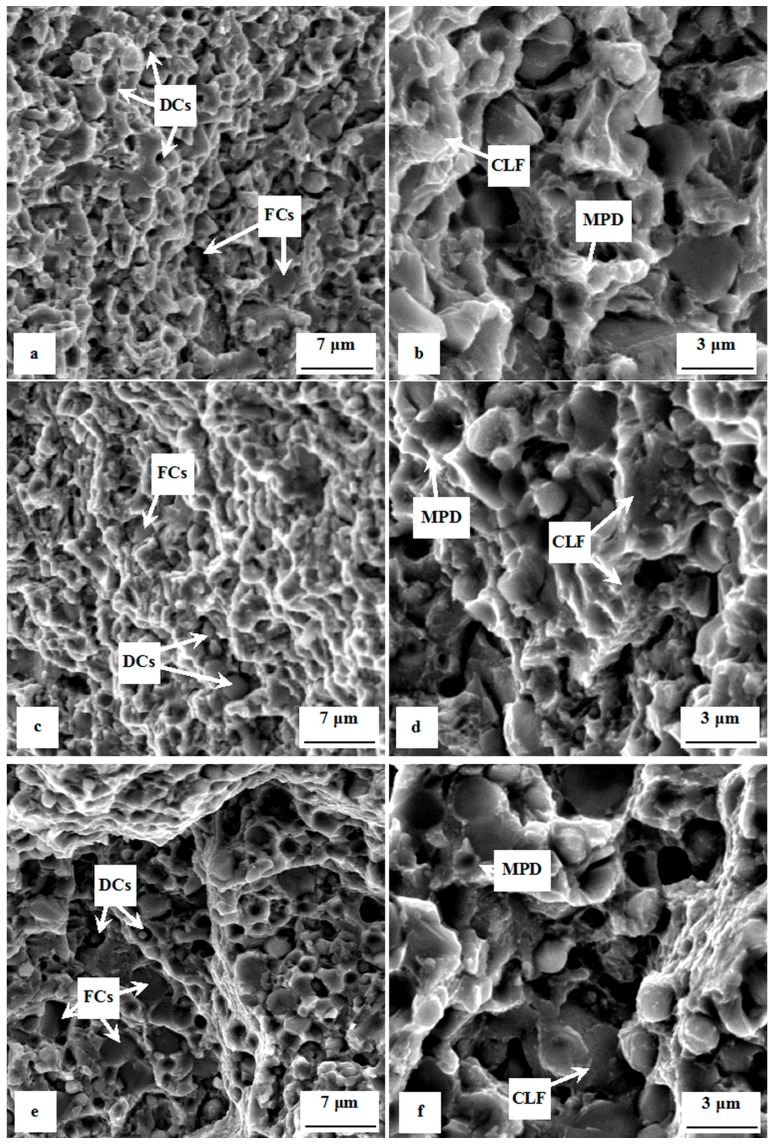
SEM micrographs showing fracture surfaces of specimens made of Vanadis 6 steel with different surface quality. (**a**,**b**) Milled (overview, detail); (**c**,**d**) fine ground (overview, detail); and (**e**,**f**) polished (overview, detail). MPD—micro-plastic deformation of the matrix, CLF—cleavage fracture, FCs—fractured carbides, DCs—carbides that assist in decohesive fracture mechanisms (decohesive carbides).

A compilation of SEM micrographs showing fractured surfaces of specimens with different surface roughness is shown in Figure 7. All the fractured surfaces manifest combined low-energetic ductile and cleavage morphology of the surface. The ductile component of the fracture is initiated mainly at the carbide/matrix interfaces, where microvoids are formed as a result of different plasticities (and stiffness) of the matrix and carbides. The carbides that assist in decohesive crack propagation (note that the formation of microvoids results from the decohesion at the phase interfaces) are denoted as DCs (Figure 7a,c,e). Coarser carbide particles undergo cleavage mechanisms of crack propagation more easily, and these carbides are marked as FCs (fractured carbides). The matrix mainly manifests so-called “dimple” morphology of fractures, which is associated with micro-plastic deformation (MPD), mainly on the sites of carbide/matrix interfaces. However, cleavage facettes (CLF) are also visible on the micrographs (Figure 7b,d,f). Even though the fractured surfaces appear very similar with respect to their morphology, the dimples at the carbide/matrix interfaces are deeper in the case of the specimens with better surface quality (polished). A comparison is shown in Figure 7b,d,f. This is due to higher levels of plastic deformation before the fracture, and is in good agreement with the results of three-point bending strength measurements (Figure 5), and also with the determination of the deformation energy (work of fracture), shown in Figure 6. These results suggest unambiguously that the surface quality plays an important role in the fracture behavior of relatively brittle materials like quenched and tempered PM ledeburitic steels.

These findings have great importance for the end-users of materials and tools, recommending them to make the surface as high quality as possible to prevent the initiation of cracks and to ensure the service reliability of tools. However, it should also be mentioned that the surface quality finish of tools can be reflected in the resulting surface roughness of workpieces made of different materials, which in turn influences their mechanical properties and durability. For instance, Bayoumi and Abdellatif [46], Javidi et al. [47], Sasahara [48], Noll and Erickson [49], and Taylor and Glancy [50] established that the fatigue strength reduces with increasing surface roughness in the cases of aluminium alloys, nickel–molybdenum alloys, and carbon steels, due to the stress concentrations generated by rough surfaces. Novovič et al. [51] stated that surface roughness values over 0.1 μm deteriorate the fatigue life on any component significantly. One can thus conclude that the tools’ surface finish quality not only influences the toughness characteristics of the tools themselves, but also the overall production quality.

### 3.3. Effect of Plasma Nitriding

**Figure 8:**
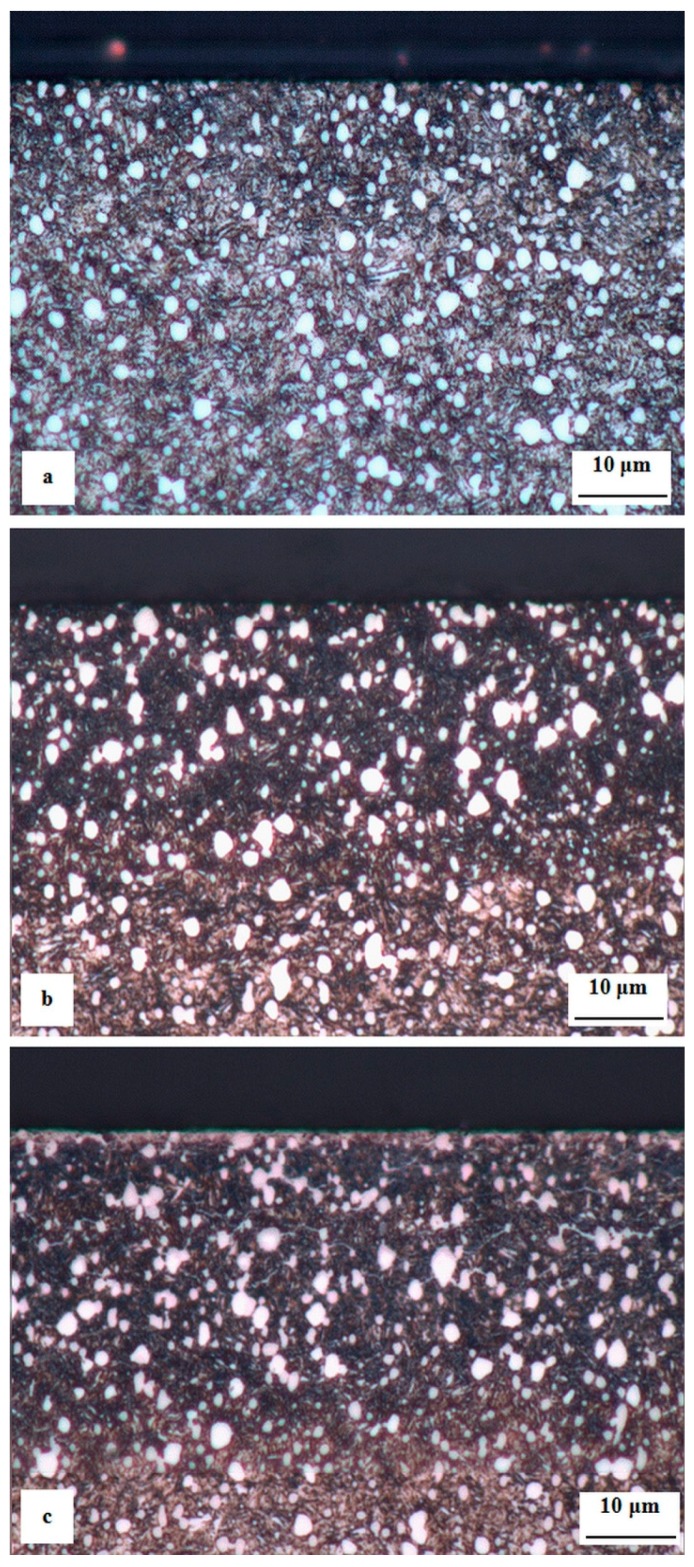
Optical micrographs showing the microstructures of differently plasma nitrided specimens: (**a**) Nitrided at 470 °C for 30 min, (**b**) nitrided at 500 °C for 60 min, and (**c**) nitrided at 530 °C for 120 min.

Figure 8 is a compilation of optical micrographs showing the microstructure of plasma nitrided regions developed on the surfaces of Vanadis 6 steel, by application of various treatment regimes. In Figure 8a, there is a nitrided region developed at the temperature of 470 °C for the duration of 30 min. The nitrided region differs from the substrate in terms of etching sensitivity, which is caused by the precipitation of nitrides. However, the nitrogen input into the material is relatively low in this case, which is reflected in only small differences in etching sensitivity between the nitrided surface and the substrate detectable in the optical micrograph. Nitriding at the temperature of 500 °C for 60 min highlights the differences in etching intensity between the nitrided region and bulk material. This is due to a higher input of nitrogen atoms into the steel substrate. However, neither the processing at 470 °C nor that at 500 °C leads to the formation of a compound “white” layer on the surface. Furthermore, the presence of nitride networks at the primary grain boundaries was not detected (Figure 8b). The micrograph in Figure 8c demonstrates that the nitriding at the temperature of 530 °C for the duration of 120 min leads to both the growth of a thin compound “white” layer on the surface (in this particular case, the white layer had a thickness of 1–1.5 μm) and formation of nitride networks at the original grain boundaries. 

**Figure 9:**
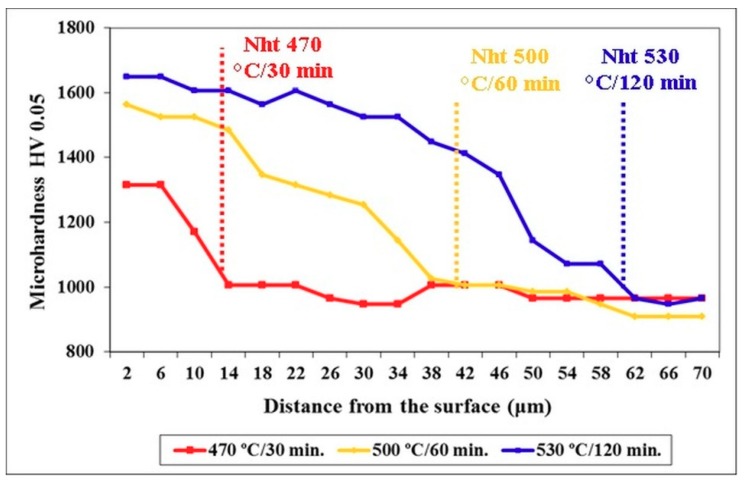
Microhardness depth profiles for differently nitrided specimens.

Table 3 summarizes the main parameters of nitrided regions. The surface concentration of nitrogen is 4.03 wt.% in the specimen nitrided at 470 °C for 30 min, and it increases moderately as the nitriding temperature is increased (or for longer processing durations). The nitrogen diffusion depths are 13.2, 42, and 67 μm for the specimens nitrided at 470, 500, and 530 °C, respectively, and these values correspond well with the values of nitriding case depth (Nht). The surface hardness values are 1316, 1564, and 1648 HV0.05 for the same specimens. In Figure 9, there are microhardness depth profiles for all these specimens. It is shown that the microhardness of the material nitrided at 470 °C for 30 min decreases rapidly with the increasing depth below the surface, while the microhardness depth profiles of other specimens appear relatively flat, with moderate decreases in the values. 

**Figure 10:**
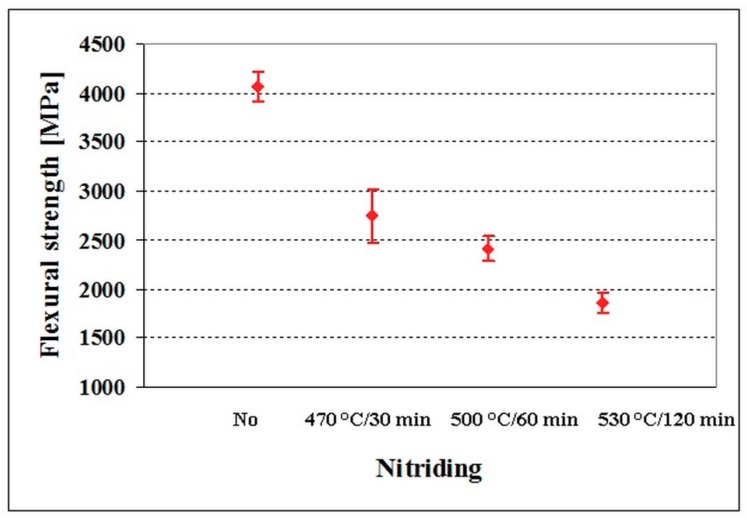
Flexural strength of non-nitrided and differently plasma nitrided specimens made of Vanadis 6 steel.

Figure 10 shows the flexural strengths of the materials as a function of plasma nitriding parameters. Non-nitrided material has the flexural strength of 4071 ± 154 MPa. The specimens nitrided at 470 °C for 30 min, 500 °C for 60 min, and 530 °C for 120 min have values of flexural strength of 2744 ± 282, 2414 ± 126, and 1861 ± 99 MPa, respectively. These results suggest that the diffusion regions on the surfaces reduce the flexural strength (and toughness) of the examined steel dramatically, and that this reduction increases with increasing the thickness of the nitrided region, its increasing saturation with nitrogen, hardness, etc.

**Figure 11:**
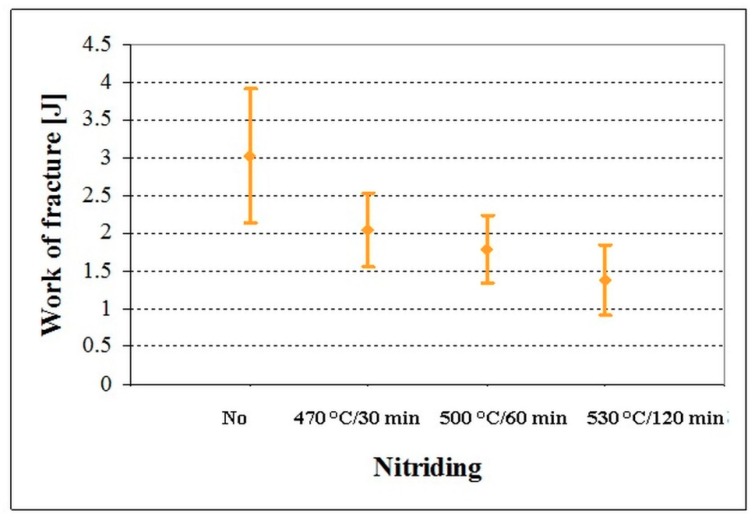
Work of fracture of non-nitrided and differently plasma nitrided specimens made of Vanadis 6 steel.

The work of fracture of the non-nitrided specimen was 3.02 ± 0.88 J (Figure 11). Considerable reduction of flexural strength (Figure 8) is reflected in reduction of work of fracture. The values of *W*_of_ were 2.03 ± 0.49, 1.79 ± 0.44, and 1.38 ± 0.47 J for the differently plasma nitrided specimens. 

**Figure 12:**
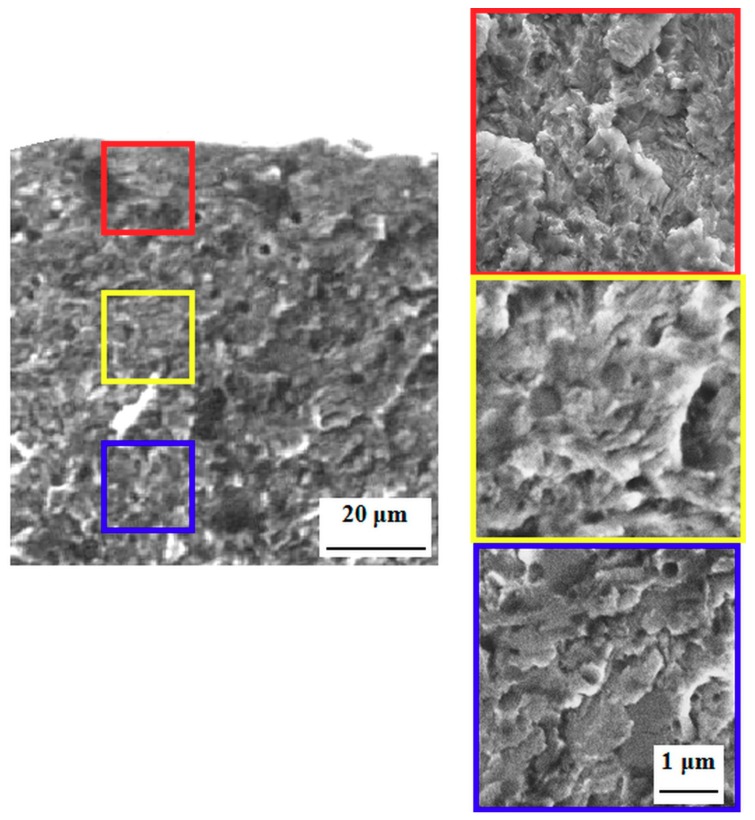
SEM micrographs showing fracture surface of specimen made of Vanadis 6 steel nitrided at 530 °C for 120 min.

Furthermore, the fracture surfaces of nitrided steel reflect clearly the reduction of flexural strength. The surfaces manifest cleavage fractures (Figure 12) and the thickness of the cleavage regions corresponds well with the thickness of the nitrided regions. The fracture propagation manner through the bulk material is influenced by the presence of the nitrided region, and the topography of the fracture is significantly reduced (compare Figure 12, which is a detailed micrograph in a blue border, with Figure 7). The cleavage propagation manner, with no indication of plastic deformation, is a typical feature of nitrided regions, and the morphology of the fractured surface does not manifest any changes in different depths below the surface (Figure 12).

The obtained results have great importance towards the industrial practice. Diffusion processes like nitriding or boronizing are widely used in industry in order to obtain benefits in hardness [7,52,53], wear performance [8,9], and corrosion behavior [10]. Furthermore, the industrial PVD coating companies often use nitriding as a pre-treatment prior to the deposition of hard ceramic layers, forming so-called duplex-coatings on the surface [11,12,13,14,15,16,17,54]. The duplex system (nitriding + PVD coating) brings many benefits to the surface treatments technology due to better support for the coating [16,54], which leads to much better adhesion of thin films to the steels [12,13,16]. However, the heat treaters must not forget the detrimental effects of the presence of nitrided layers on the toughness. In the work [55], it has been demonstrated that the flexural strength was significantly reduced not only for thin specimens, but that this reduction is practically independent of the specimen cross-section size. Hence, the nitriding processes should be controlled carefully in order to avoid the formation of either too thick nitrided regions or continuous networks of nitrides along the grain boundaries, and to maintain at least acceptable material toughness. 

### 3.4. Effect of PVD Coating with Cr_2_N-6Ag

**Figure 13:**
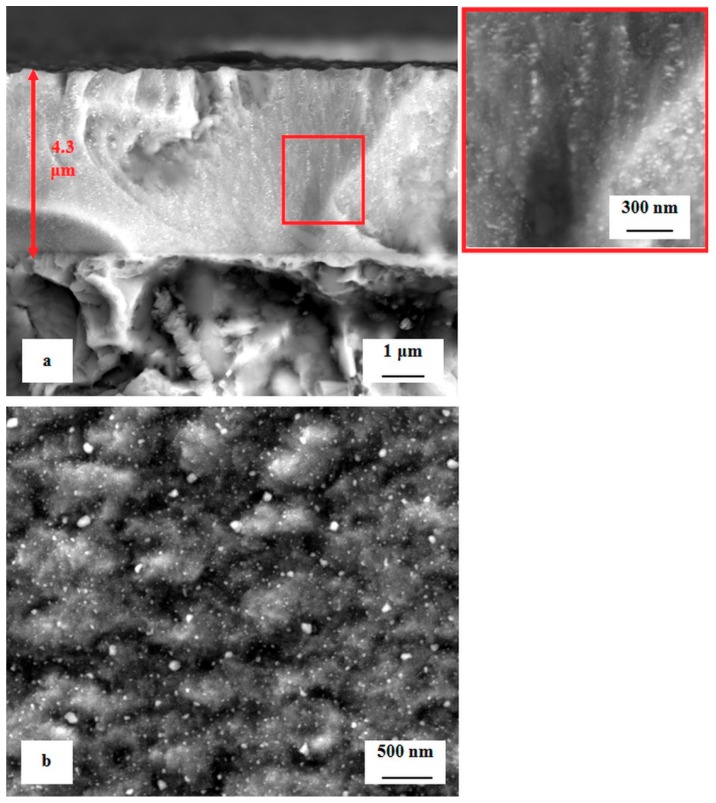
Microstructure of Cr_2_N–6Ag film deposited on the Vanadis 6 tool steel. (**a**) Cross-sectional SEM micrograph, overview, detail; (**b**) plan-view SEM micrograph.

The thickness of Cr_2_N film with an addition of 6 mass% Ag is 4.3 μm, Figure 13. The film grew in a columnar manner, and the individual crystallites are well visible in the SEM micrograph. This growth manner is typical for magnetron-sputtered Cr_2_N films developed at the same or similar combinations as the processing parameters [56,57,58]. Furthermore, it is shown in Figure 13a that silver forms individual agglomerates within the Cr_2_N. The reason for this is that the silver is completely insoluble in CrN, as reported recently [39,40,41,42]. The plan-view SEM micrograph (Figure 13b) illustrates that the sizes of agglomerates range between several nm and 60 nm, and that the coarser Ag particles are located mainly between the individual Cr_2_N crystallites, while the finer ones can also be found within the crystallites of Cr_2_N.

The mechanical properties of the Cr_2_N–6Ag film are summarized in Table 4. As reported recently [39,41], the addition of silver does not influence the mechanical properties negatively in comparison with pure Cr_2_N. This was attributed to the grain refinement caused by presence of Ag in the Cr_2_N compound, which more than sufficiently compensated the fact that both the hardness and the Young modulus of the silver are much lower than the values normally obtained for pure Cr_2_N.

**Figure 14:**
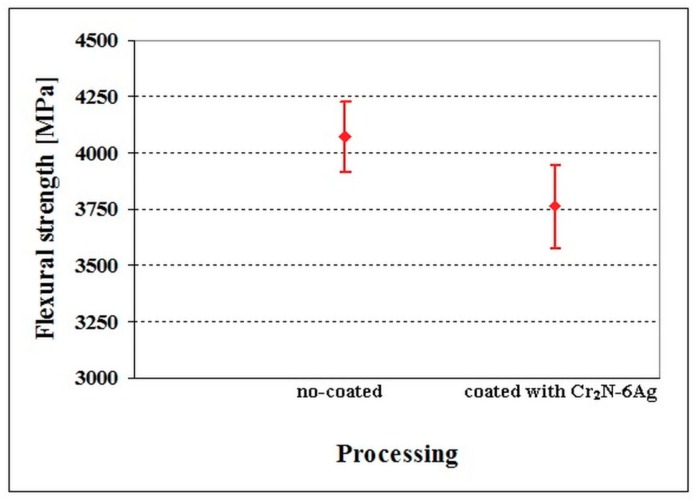
Flexural strength of no-coated and Cr_2_N–6Ag-coated Vanadis 6 steel. The values of flexural strength are adapted from the corresponding literature [59].

**Figure 15:**
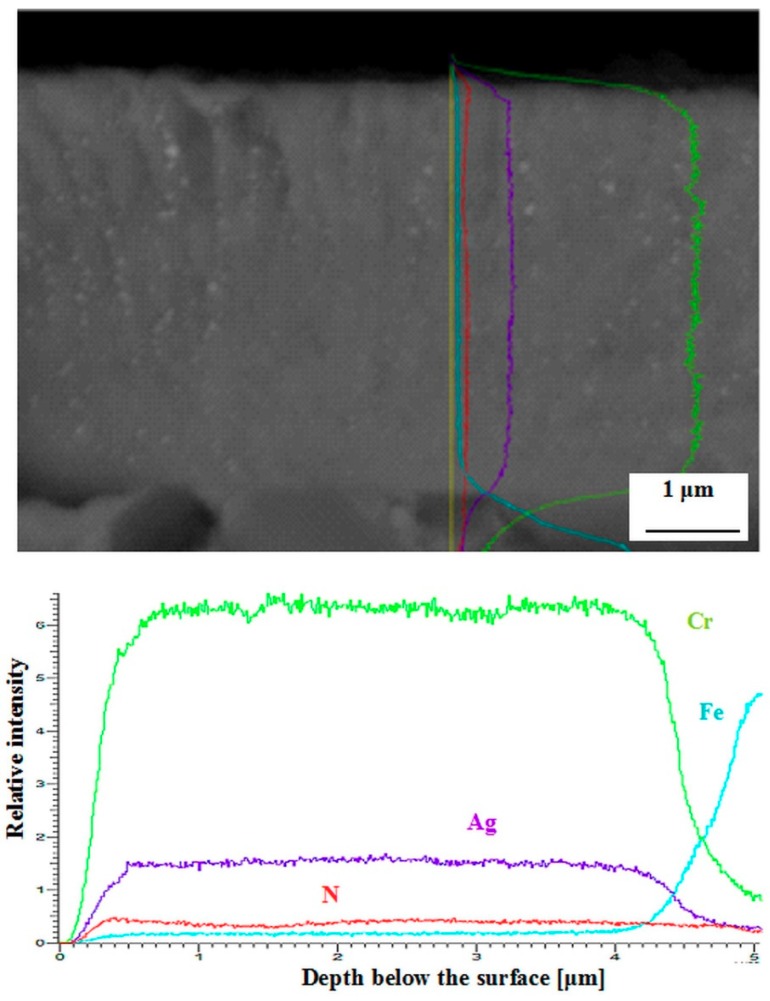
Concentration depth profiles of iron and the main coating elements, indicating a non-diffusive coating/substrate interface.

**Figure 16:**
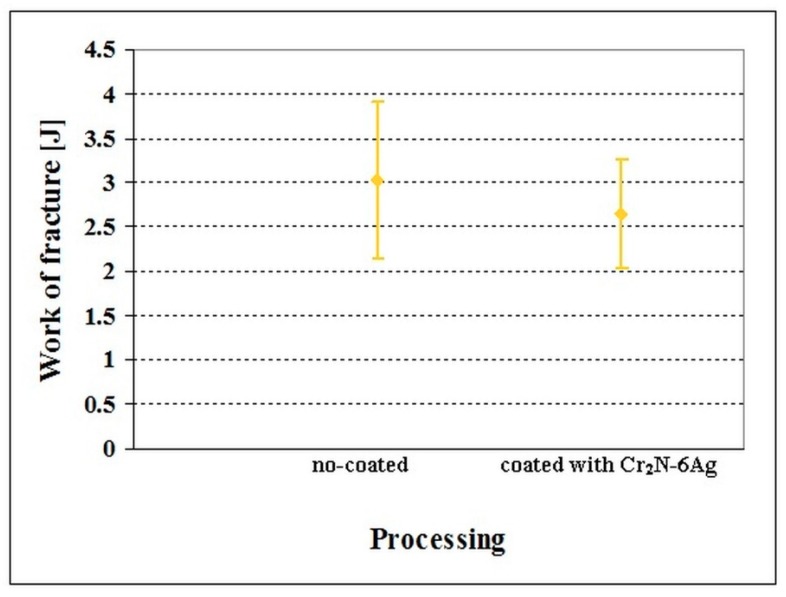
Work of fracture of no-coated and Cr_2_N–6Ag-coated Vanadis 6 steel. The values are adapted from the corresponding literature [59].

**Figure 17:**
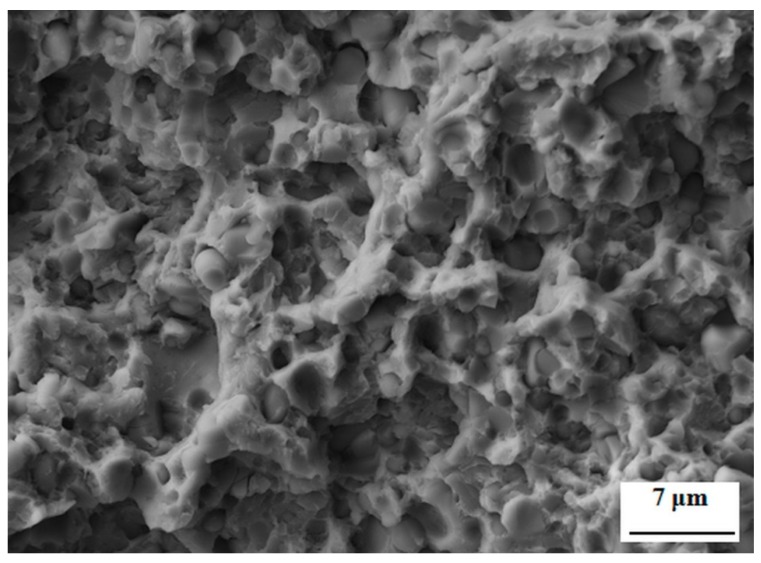
SEM micrographs showing the fracture surface of Cr_2_N–6Ag-coated Vanadis 6 steel.

The flexural strength values of no-coated and Cr_2_N–6Ag-coated Vanadis 6 steel manifested only marginal differences (Figure 14). The flexural strength of no-coated specimens was 4071 ± 154 MPa, while that of coated ones was 3760 ± 185 MPa. This is because there is almost no diffusion interface between the substrate and the coating, as indicated in Figure 15. Insignificant differences in flexural strength values are reflected in the values of work of fracture (Figure 16). Even though the mean value of no-coated specimens seems to be slightly higher than what was obtained for the coated steel, the statistical uncertainty ranges for both material states considerably overlap, suggesting that the work of the fracture is only slightly influenced by the presence of a PVD coating. The mentioned results are also reflected in the fracture surfaces of the Cr_2_N–6Ag coated specimens. The fracture surface manifests well-visible dimple morphology, with local micro-plastic deformation at the carbide/matrix interfaces (Figure 17). This propagation manner is typical for hard materials like tool steels, as reported recently [3], and is associated with enhanced fracture surface roughness and an increase of the ductile microvoid coalescence micro-mechanism in fracture surfaces for these microstructures. One can also claim that the fracture surface of Cr_2_N–6Ag coated steel does not differ significantly from the ones obtained by the flexural strength testing of no-coated steel (Figure 5).

Even though a huge number of tools are coated with thin ceramic films over Europe, the information on their damages due to presence of these films on their surfaces is lacking. Furthermore, a personal experience of the author of the current paper indicates that coated tools made of ledeburitic and/or high speed steels are damaged due to poor steel quality rather than as a result of the coating presence on the surface. One can thus suggest that the coaters as well as the end-users of coated tools do not risk tool embrittlement due to PVD coating, and that the only risk for tool damage is the choice of the substrate material with poor metallurgical quality. 

## 4. Conclusions

The obtained and summarized results on the effect of different surface engineering techniques on bulk toughness of hard and brittle Cr–V ledeburitic tool steel Vanadis 6 unambiguously indicate that:

Enhanced surface roughness has unfavorable impacts on the toughness of the material, and thereby on the durability of tools, particularly when they are heavily and dynamically loaded. It should be underlined that elevated surface roughness of tools is transmitted to the surfaces of workpieces, which leads to worsened properties of workpieces.

The presence of diffusion nitrided regions on the steel surface reduces the toughness of the material considerably. The detrimental effect of nitrided regions is evident particularly in the cases when either a surface “white” layer is formed or continuous nitrides appear at the boundaries of primary austenite grains.

PVD coating has almost no diffusion, and hence, its effect on the material toughness can be classified as marginal. Hence, the risk of material embrittlement due to the deposition of PVD thin films is minimal.

The abovementioned facts have clear consequences for the designers, heat treaters, and end-users of the tool products. While the presence of thin ceramic films does not influence the mechanical properties of the tools negatively, the enhanced surface roughness as well as the presence of nitrided regions reduces the material toughness. In nitriding, therefore, the heat treaters must inevitably avoid the formation of too thick regions, or reduce the probability of the growth of compound “white” layers on the surface. The tool designers, on the other hand, should keep in mind that the surface finish quality (roughness) influences not only the toughness of the tool itself but also the quality of the workpieces.

## Figures and Tables

**Table 1:** Characterization of specimen batches used in the experimental works.

Batch	Surface Finish	Post Heat Treatment	Total Number of Specimens
1	Milled, R_a_ = 6 μm	No	5
2	Ground, R_a_ = 0.3 μm	No	5
3	Mirror finish polished, R_a_ = 0.017 μm	No	5
4	Mirror finish polished, R_a_ = 0.017 μm	Plasma nitriding	15
5	Mirror finish polished, R_a_ = 0.017 μm	Coating with Cr_2_N-6Ag	5

Batches 1, 2, and 3 were examined without any post-heat treatment. Batch 4 was subjected to the plasma nitriding, and the specimens from Batch 5 were coated with a Cr_2_N–6Ag nanocomposite thin film.

**Table 2:** List of plasma nitriding parameters applied for the treatment of specimens.

Batch	Temperature (°C)	Duration (min)
4.1	470	30
4.2	500	60
4.3	530	120

**Table 3:** Main parameters of nitrided regions (*the nitriding case depth Nht is determined as core hardness + 50 HV0.05).

Nitriding Parameters	Surface Nitrogen Content (wt.%)	Nitrogen Diffusion Depth (μm)	Surface Microhardness (HV 0.05)	Nht* (μm)
470 °C/30 min	4.03	13.2	1316	13
500 °C/60 min	4.8	42	1564	39
530 °C/120 min	5.78	67	1648	60

**Table 4:** Mechanical properties of the developed Cr_2_N–6Ag coating.

Coating	Nano-hardness (GPa)	Young´s Modulus (GPa)
Cr2N-6Ag	16.17 ± 1.93	263 ± 17
